# Acoustic stimulation and other emerging approaches to enhance sleep: design notes for the next generation of closed-loop neurostimulation technology

**DOI:** 10.3389/fnins.2025.1682450

**Published:** 2026-02-04

**Authors:** William G. Coon, Sophie J. Nilsson, Michael T. Smith, Matthew J. Reid

**Affiliations:** 1 Johns Hopkins University Applied Physics Laboratory, Intelligent Systems Center, Laurel, MD, United States; 2 Johns Hopkins University, G.W.C. Whiting School of Engineering, Baltimore, MD, United States; 3 Department of Psychiatry and Behavioral Sciences, Johns Hopkins School of Medicine, Baltimore, MD, United States

**Keywords:** sleep, acoustic stimulation, closed-loop stimulation, neurostimulation, spindles, slow wave sleep (SWS), slow oscillation

## Abstract

Sleep is indispensable to human health, supporting memory consolidation, emotional regulation, immune function, and metabolic homeostasis. Despite its importance, chronic sleep disturbances are pervasive, with especially high prevalence in operational and clinical populations. This review synthesizes recent advances in sleep enhancement through closed-loop neurostimulation, focusing on systems that dynamically interact with endogenous brain rhythms to improve sleep quality and efficiency. Key oscillatory targets—including slow waves, sleep spindles, and hippocampal ripples—are examined in the context of memory consolidation, with evidence supporting their augmentation via temporally precise auditory and electrical stimulation. Complementary methods targeting rapid eye movement (REM) sleep and sleep onset latency are discussed, underscoring the versatility of closed-loop systems. The review identifies outstanding questions regarding stimulation timing, modality selection, physiological limits, and the dissociation between slow oscillations and delta activity. To address these challenges, we advocate for a modular, open-source ecosystem that integrates real-time sleep state decoding with configurable effectors across auditory, electrical, and other domains. Such a platform would enable reproducible, scalable, and personalized interventions for sleep enhancement. This systems-level approach is aimed at accelerating translational research and catalyzing a paradigm shift toward actively regulated, on-demand sleep interventions.

## 1 Need

Sleep is fundamental to good health and optimal performance, yet many Americans have chronically disrupted sleep (Krueger and Friedman, 2009), especially those in the US armed services (Mysliwiec et al., 2013; Luxton et al., 2011). Sleep that is insufficient in duration or quality reliably impairs cognitive function (Killgore, 2010), learning capacity (Curcio et al., 2006), memory (Newbury et al., 2021; Diekelmann and Born, 2010), immune system function (Bryant et al., 2004), neural waste metabolite clearance (Xie et al., 2013), physical recovery (Rae et al., 2017), cardiovascular health (Mullington et al., 2009), and metabolism (Knutson et al., 2007). Concerningly, it has been linked to cognitive decline and poor quality of life in old age (Scullin and Bliwise, 2015), deposition of amyloid plaques, dementias including Alzheimer's Disease, (Spira et al. 2014), and may even underlie the increased risk of these neurodegenerative sequelae in those who have suffered a traumatic brain injury (TBI) (Piantino et al., 2022; Peters and Lyketsos, 2023; Hablitz and Nedergaard, 2021). Although many of the functions above have been linked to non-rapid eye movement (NREM) sleep, a degree of evidence suggests rapid eye movement (REM) sleep plays a critical role in processing emotional memory (Van der Helm and Walker, 2011; Wassing et al., 2019), and dysfunction of this process may contribute to post-traumatic stress disorder (PTSD) (Rho et al., 2023; Mellman et al., 2002). Both NREM and REM sleep are crucial for optimal health (Lubin et al., 1974).

Systems that can automatically initiate and maintain different sleep states would offer whole new avenues to treat disease (Geiser et al., 2020; Zhang and Gruber, 2019; Fröhlich and Lustenberger, 2020), and may one day be able to increase the restorative efficiency of sleep, achieving sleep benefits in less time.

This manuscript reviews current approaches to enhance sleep through closed-loop neurostimulation with a focus on how the physiology of sleep, problem-space constraints, and emerging technologies can inform the design of next-generation sleep enhancement platforms. Many excellent and comprehensive reviews have already been published on the effects of sleep neurostimulation. To complement this literature, this manuscript instead synthesizes key insights to outline principles for advancing the closed-loop technologies that support research in this space.

**Terminology and band definitions used in this review**. To harmonize usage across sections: we use slow oscillation (SO) to denote ~0.5–1.25 Hz; delta (δ) to denote 1–3 Hz; and slow wave activity (SWA) to denote the broad 0.5–4 Hz power band commonly used in sleep research (noting that AASM/R&K scoring historically emphasize waves < 2 Hz). For spindles we refer to slow (11–13 Hz) and fast (13–16 Hz) subbands when relevant; for hippocampal ripples we note that human frequencies are variable (often 80–200 Hz) and broader than in rodents. Phase descriptions below use this convention.

## 2 Target mechanisms for engineering enhanced sleep

**Figure 1:**
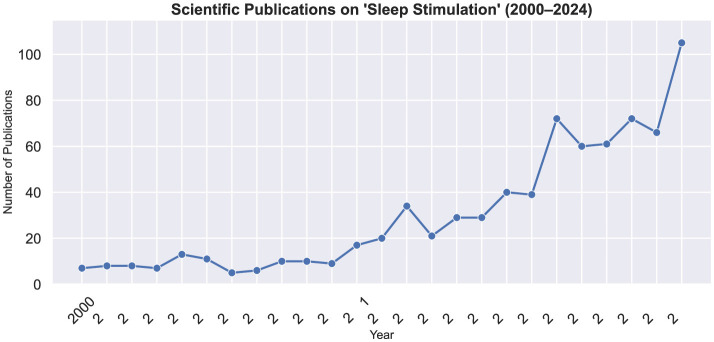
Increasing interest in sleep stimulation. Graph shows the number of scientific publications on sleep stimulation over 25 years. Note the rapid increase in the number of annually published studies on sleep stimulation beginning in the 2010–2011 time frame. Data derived from OpenAlex.org on June 24, 2025, using results aggregated from the following six independent queries: [“acoustic stimulation sleep,” “auditory stimulation sleep,” “transcranial stimulation sleep,” “electrical stimulation sleep,” “thermal stimulation sleep,” “slow wave stimulation sleep”]. Results were not filtered for conference proceedings but duplicates were removed.

The past 15 years have witnessed a rapid acceleration in the number of papers published on stimulating the brain during sleep (Figure 1). Many of these have used closed-loop neurostimulation^1^ to increase specific physiological mechanisms during sleep, with concomitant increases on specific functions (e.g., memory). These protocols are mature and well-validated but may not be sufficient to meaningfully increase the amount of time spent in a specific sleep state (e.g. REM or NREM sleep). Nevertheless, some emerging techniques have begun to show promise for both slow wave sleep (SWS) and REM sleep enhancement. Importantly, they are not all mutually exclusive and—with the right technology—many could be deployed in parallel.

### 2.1 Enhancing specific physiological mechanisms and functions during sleep

Much of what we know about how to interact with the brain during sleep to improve human performance comes from learning and memory studies. Decades of research have revealed the neurophysiological substrates of sleep-mediated memory processing. The three best-understood electrophysiological oscillations involved in this process (slow waves, ripples, and sleep spindles) have proven amenable to augmentation.

#### 2.1.1 Physiological mechanisms of sleep-mediated learning and memory

**Figure 2:**
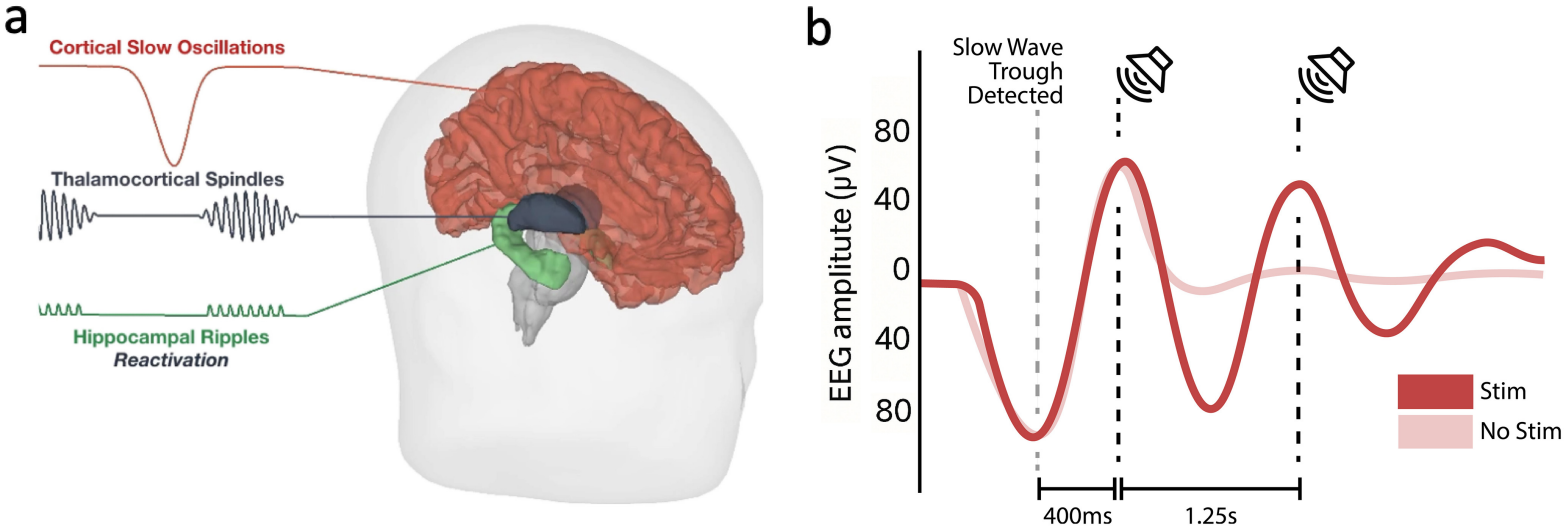
Physiological targets for closed-loop neurostimulation during sleep. In **(a)**, three nested oscillations that mediate memory consolidation during NREM sleep. Their interplay typically begins with a cortically synchronized hyperpolarizing burst, which is reflected in the sharp downward deflection of the slow oscillation's inhibitory “down” state. The down state is followed by an excitatory “up” state lasting several hundred milliseconds. During the upstate rebound, sleep spindles, which are propagated through thalamocortical circuits, are thought to bind distant cortical regions together to facilitate the consolidation from short-term memory stores dependent on support from hippocampal circuits to long-term stores in cortex that no longer require hippocampal scaffolding. The entrainment of widespread cortical areas by spindles may support the integration of newly formed memories with prior experience, context, and skills acquired by the organism throughout its life. Hippocampal ripples are thought to reflect this outward flow of information from the hippocampus to the cortex. Ripples nest in the troughs of spindle oscillations. Isolated ripple events may reflect a strengthening of synaptic connections (memory consolidation), while sequentially occurring bursts of ripple doublets or triplets may support pattern completion (memory post-processing). In summary, triple phase locking between cortical slow oscillations, thalamocortical spindles, and hippocampal ripples is thought to mediate memory processing during (NREM) sleep. This circuit system is highly amenable to augmentation via closed-loop neurostimulation during sleep. In **(b)**, a typical closed-loop neurostimulation protocol to enhance slow wave activity. The down state (trough) is detected in real time and stimuli (auditory or electrical) are timed to follow during subsequent up states to increase slow wave activity.

Sleep is critical for learning and memory (Newbury et al., 2021; Diekelmann and Born, 2010; Rasch and Born, 2013). Some of the most well understood physiological mechanisms supporting sleep's information processing benefits are amenable to modulation by neurostimulation. During sleep, transient synchronization of the hippocampus and cortex supports the transfer of new learning from short-term (hippocampus-dependent) memory to long-term (cortex-dependent, hippocampus-independent) memory. This synchrony is orchestrated by the interplay of nested oscillations at different temporal scales (Figure 2), including hippocampal ripples (80–200 Hz), thalamocortical spindles (13–16 Hz), and cortical slow waves (0.5–4 Hz). Ripples coincide with the reactivation of new learning encoded in hippocampal circuits during non-rapid eye movement (NREM) sleep (Wilson and McNaughton, 1994; Lee and Wilson, 2002; Davidson et al., 2009; Siapas and Wilson, 1998), correlate with overnight memory consolidation, and co-occur with sleep spindles, cortical rhythms visible in the scalp electroencephalogram (EEG) as waxing/waning oscillations (Clemens et al., 2011, 2007; Staresina et al., 2015; Latchoumane et al., 2017; Coon et al., 2019). Spindles are also believed to play a causal role in memory consolidation (see Manoach and Stickgold, 2019, Rasch and Born, 2013, and Fogel and Smith, 2011 for reviews). Spindle-ripple coupling is further organized by cortical slow waves (Staresina et al., 2015; Latchoumane et al., 2017), which are bursts of hypersynchronous neuronal activity that traverse the cortex during NREM sleep (Massimini et al., 2004). Spindle-ripple coupling occurs in the prolonged voltage-positive “up” state of a slow wave, as the EEG voltage potential rebounds from the slow wave's onset-defining negative “down” state (Staresina et al., 2015; Helfrich et al., 2018). Since cortical neurons are more excitable during up states, slow waves may serve to initiate a temporal “reading frame” for memory replay in which hippocampal reactivation of recent learning can propagate to the cortex when it is most receptive to afferent input. The slow wave's role in this sequence of oscillatory events has made it a conspicuous target for neurostimulation to enhance memory.

#### 2.1.2 Enhancing memory by augmenting slow oscillations, spindles, and ripples

Numerous studies have shown that memory can be enhanced by increasing slow waves, spindles, and ripples directly via electrical or auditory stimulation during sleep (Ngo et al., 2013; Geva-Sagiv et al., 2023; Lustenberger et al., 2016). A recent meta-analysis and review by Wunderlin et al. (2021) highlights a principle that applies broadly to sleep neurostimulation: timing matters. For slow wave stimulation, stimuli must be delivered during NREM sleep after the onset of an endogenous slow wave has been detected and during its excitable up state (approximately 300–500 ms after detecting an onset down state^2^) (Ong et al., 2016). Indiscriminately stimulating during NREM sleep appears to have no effect on memory (Weigenand et al., 2016), pointing to the necessity of phase precision from closed-loop detector/stimulator setups with real-time processing capabilities to enhance memory via slow wave stimulation.

Interestingly, auditory stimulation (often brief stimuli, such as a 50 ms bursts of pink noise) has been shown to be as effective as electrical stimulation (e.g. transcranial electrical stimulation or tES) at enhancing memory (Ong et al., 2016; Papalambros et al., 2017)^3^ and increasing slow wave activity, at least in younger adults (Wunderlin et al., 2021; Fehér et al., 2021; Ngo et al., 2012; Debellemanière et al., 2021) [however, tES may be more effective at increasing slow waves irrespective to any effect on memory (Dondé et al., 2021)]. Auditory stimulation is often preferred because it does not produce the same electrical artifact during stimulation periods that electrical stimulation does. tES artifact can obscure brain activity measurements for up to several seconds post-stimulation (Noury and Siegel, 2017).

Sleep spindles have also been enhanced with closed-loop neurostimulation (Lustenberger et al., 2016; Choi et al., 2019; Baxter et al., 2023), as have hippocampal ripples (Geva-Sagiv et al., 2023), making all three oscillatory phenomena viable targets for neurostimulation during sleep to enhance performance. However, only two of these oscillations—spindles and slow oscillations—are accessible from the scalp; ripple stimulation requires techniques that can reach deep brain structures like the hippocampus through e.g., deep brain electrical stimulation (which requires surgery), as well as higher temporal precision and frequency resolution for their detection. As discussed further in Section 4: Effectors, there is some evidence that advanced non-invasive strategies such as spatially focused Temporal Interference (TI) can provide a non-invasive alternative.

Most studies using closed-loop auditory stimulation (CLAS) have attempted to enhance memory consolidation during sleep. Many of the overarching trends found in these studies are consistent and well replicated. Nevertheless, there continue to be persistent conflicts in study outcomes. For example, studies of declarative memory have found a significant improvement in word-pair retention after undergoing closed-loop auditory stimulation during a full night of rest (Ngo et al., 2013, 2015; Leminen et al., 2017; Papalambros et al., 2017), a finding that has also been replicated in naps (Ong et al., 2016). However, in other recent studies this result could not be replicated in all male (Harrington et al., 2021) or older adult (ages 49–63) (Schneider et al., 2020) samples. A study by Henin et al. (2019) found no significant difference in word-pair retention between acoustic stimulation and sham conditions in both nap and overnight trials. Another recent study did not find an improvement in word-pair, figure-pair, or verbal task performance after acoustic stimulation during a nap (Koo-Poeggel et al., 2022). Procedural memory has also been shown to benefit from stimulation (Lustenberger et al., 2016; Choi et al., 2019; Choi and Jun, 2022), but not universally (Baxter et al., 2023).

#### 2.1.3 Challenges targeting physiological transients instead of sleep states

These techniques can only increase sleep waveforms up to a point, and the process appears to be self-limiting (Ngo et al., 2015). Continuous presentation of stimuli—even just to slow wave “up” states—does not increase the effect compared to one or two stimuli post-detection of an endogenous slow wave (Fehér et al., 2021), and the increase in slow wave activity (SWA) or amplitude tends to be strongest during the first 1-3 stimuli, and then diminishes with each subsequent consecutive stimulus (Bellesi et al., 2014), consistent with a self-limiting process. Spindle augmentation has similarly been shown to be subject to a refractory period (Lustenberger et al., 2016). Hence, other techniques may be required to increase time spent in specific stages e.g., slow wave sleep, which is characterized by long periods of continuous slow wave activity (i.e., many more slow waves than above techniques can provide).

#### 2.1.4 Practical burst scheduling

To maximize the likelihood of a robust response in a manner consistent with refractory/self-limiting dynamics, we recommend delivering at most 1–2 pulses per detected SO up-state. We also recommend limiting consecutive detections to 3–5 slow waves before imposing an inter-train “quiet” window of 10–20 s, and duty-cycling stimulation blocks across the night (e.g., alternating 3–5 min ON/3–5 min OFF). To avoid inducing unwanted awakenings or arousals while maximizing stimulus effectiveness, per-subject titration of volume is advised. These heuristics reflect empirical response fall-offs and refractory periods observed across studies (Bellesi et al., 2014; Lustenberger et al., 2016; Fehér et al., 2021; Debellemanière et al., 2021).

#### 2.1.5 How precise is “precise enough” for effective closed-loop timing?

In practice, a wide variety of modern detector techniques achieve sub-cycle precision adequate for SO up-state targeting. Fixed-delay schemes (e.g., ~300–500 ms after a detected negative peak) are common and effective. Nevertheless, inter-individual variability in intrinsic SO period may motivate adaptive timing (subject-specific delay/phase) and online recalibration (such adaptations are increasingly standard). There is ample time for online signal processing, such as analytic signal derivation for the estimation of instantaneous phase, in modern hardware. Reported end-to-end latencies (acquisition + processing + audio output) are commonly on the order of < 100–150 ms on research-grade systems, with phase-targeting errors on the order of a few tens of degrees for SOs—sufficient for robust SO/SWA augmentation in young adults (Ngo et al., 2013; Ong et al., 2016; Fehér et al., 2021). Although both fixed-delay and adaptive phase-tracking algorithms (e.g., “phase-locked loop” (PLL) or Hilbert-based instantaneous phase) are used to time acoustic stimuli to the SO up-state (Ngo et al., 2015; Santostasi et al., 2016), we found no head-to-head human studies directly comparing these approaches on physiological and behavioral outcomes and it is not yet clear whether additional phase precision translates to increased effectiveness. An engineering evaluation using re-streamed human EEG favored a simple fixed-step rule over a PLL implementation for reliability (Piorecky et al., 2021), while phase-mapping studies indicate a relatively tolerant “rising-phase” window—especially in younger adults—implying that well-tuned fixed-delay rules can suffice in many settings, with tighter phase targeting potentially more valuable in older or low-SWA cohorts (Navarrete et al., 2020).

### 2.2 Enhancing specific sleep states

The challenges with modulating entire sleep states (REM, NREM) are illustrated by a matter of scale—to date, the most effective techniques to modulate the sleeping brain have targeted oscillatory transients that unfold over hundreds of milliseconds; sleep states unfold over tens of minutes to hours. Increasing a few transients is insufficient to alter sleep at the scale at which it unfolds. However, one principle is likely to hold across these different temporal domains: timing matters. The effectiveness of closed-loop neurostimulation targeting e.g., slow waves, has been shown to be highly dependent on proper timing (see above and Outstanding Questions below). Sleep state modulation is also likely to be most effective when properly timed to slower ultradian rhythms like sleep cycles.

**Figure 3:**
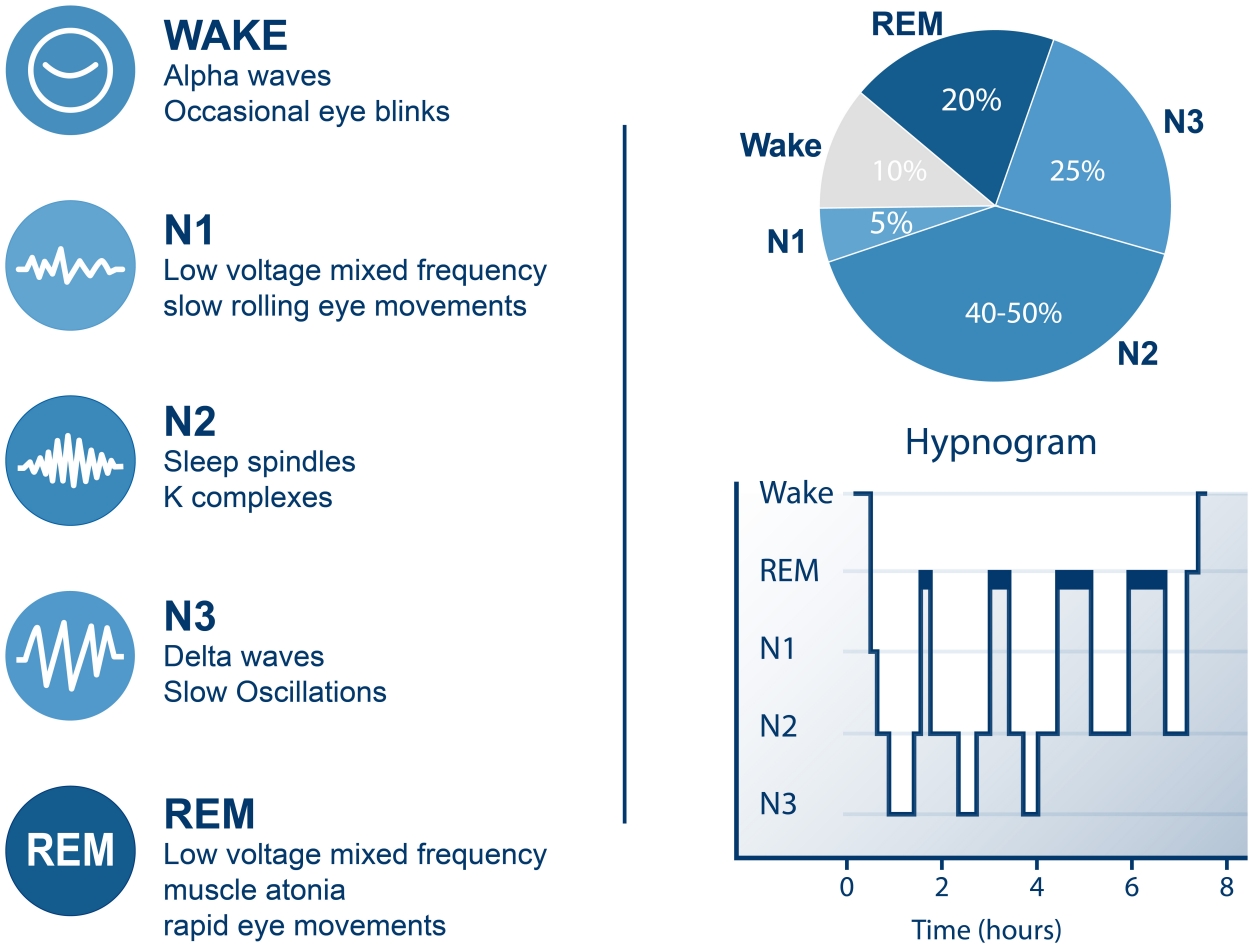
Sleep cycles through NREM and REM stages multiple times per night. Healthy sleep progresses from wakefulness through increasingly deep stages on NREM sleep before entering REM sleep. This cycle unfolds over approximately 70–120 min. As the night progresses, each cycle exhibits decreasing amounts of deep NREM sleep (N3 or slow wave sleep (SWS)) and increasing amounts of REM sleep.

Over the course of a night, healthy human sleep cycles every approximately 90 min (Carskadon et al., 2005), stereotypically transitioning through light to deep NREM sleep and then REM sleep before either beginning another cycle or terminating in wakefulness (Figure 3). This progressive stage order (N1>N2 > SWS > REM) may be disturbed by pathological (e.g., Narcolepsy, Parkinson's Disease) or pharmacological influences, and as such should not be considered a fixed cascading process but a default pattern in normal healthy sleepers. Nevertheless, this rhythmicity may be exploited to time interventions to occur within prescribed temporal windows synced to the sleeper's own endogenous sleep cycles. Clinical populations may prove more challenging. Taking full advantage of this rhythmicity requires online processing to decode the sleeper's sleep state in real time. While accurate retrospective decoding of sleep states from clinical PSG has now become trivial and commonplace (see Perslev et al., 2021 for an excellent example), fully automated and equally accurate sleep stage decoding from compact wearable EEG devices presents additional challenges—most prominently the increased presence of artifact and signal dropout inherent to wearable forehead EEG—for which few widespread solutions exist [although some publicly available solutions are beginning to emerge (Coon et al., 2025b)].

#### 2.2.1 Methods to modulate deep, restorative NREM or slow wave sleep (SWS)

Slow wave sleep (SWS), a term that maps well to today's definition of N3 sleep (Silber et al., 2007), is tightly coupled to homeostatic sleep need and generally considered the most restorative type of sleep (Dijk, 2009; Tononi and Cirelli, 2006). Prolonged periods of wakefulness or sleep deprivation are followed by rebounds of extended SWS to recover. A way to increase the amount of time spent in SWS during a given period of time asleep might allow sleep's most fundamental restorative effects to be achieved in less time overall, although this remains only a hypothesis at present.

The most widely-replicated method to boost sleep's slow wave activity is closed-loop neurostimulation time-locked to the sleeper's ongoing brain activity (Ngo et al., 2013; Wunderlin et al., 2021; Fehér et al., 2021; Besedovsky et al., 2017). However, the increased number of slow waves can translate to—at best—only a marginal increase in slow wave sleep. By contrast, mild, cyclic manipulation of skin temperature increased SWS overall, and disproportionately in the second half of the night, in one crossover study (*n* = 24 including three populations: young adults, elderly without sleep complaints, and elderly with primary insomnia) by Raymann and colleagues (Raymann et al., 2008). In that sample, the relative amount of second-half SWS approximately doubled compared to control, without the cyclic skin temperature fluctuations disturbing normal core body temperature; absolute minutes remained modest, likely because SWS is typically scarce late in the overnight sleep period. A related approach leveraging high heat-capacity bedding with mild cooling during sleep increased SWS by ~16% in another small study (Kräuchi et al., 2018). These findings—and the abolishment of exercise-related SWS effects when core warming is prevented (Horne and Shackell, 1987)—suggest that the *temporal gradient* between core and periphery may be the critical factor, rather than any single target temperature. It is possible that multiple heating-then-cooling cycles could, if timed to the sleeper's own NREM-REM sleep cycles and circadian phase, further enhance SWS without disturbing the sleep cycle.

#### 2.2.2 Population moderators

When interpreting results or designing future studies, special attention should be paid to the populations studied in sleep stimulation experiments. For example, older adults show reduced responsiveness to CLAS, plausibly due to lower SO amplitude/density observed in old age, and altered SO-spindle coupling (Helfrich et al., 2018). This can at least partially be mitigated by experimental protocols that employ per-subject amplitude thresholds and adaptive timing (Schneider et al., 2020) or techniques that combine multiple approaches to augment SWA, but additional attention to potential adverse events like stimulus-triggered arousals is warranted. Generalizability beyond healthy young adults remains an active area of research, and protocol parameters (thresholds, refractory windows, duty cycles) should be adapted to physiology and tolerance (see Section 2.3 and Table 1).

**Table 1:** Safety, tolerability, and practical contraindications by modality.

**Modality**	**Typical AEs/practical risks**	**Monitoring and mitigation**	**Common exclusions**
Auditory (CLAS)	Micro-arousals if too loud; rare ear fullness; tinnitus risk low at near-threshold levels	Per-subject thresholding; volume caps; ≥10-20 s refractory; duty cycling; overnight arousal logs	Active otologic disease; unmanaged tinnitus
tES/tACS	Tingling/itch/erythema; rare headache; EEG artifact windows	Limit current density/duration; gel and skin checks; artifact-aware scheduling	Epilepsy/seizure risk; implants; skull defects
TMS	Scalp discomfort; rare vasovagal/seizure	Lab setting; medical oversight; conservative trains	Seizure risk; implants; metallic cranial objects
Thermal	Hot spots/chill if control fails; thermoregulation stress	Small Δskin (< 1°C); rate limits; redundant temp sensors	Neuropathy; fever; dysautonomia
fUS	Transient warmth/paresthesia; skull heating risks	Validated low-intensity parameters; duty cycle limits	Cranial defects; implants near beam
TI	Cutaneous sensations similar to tES; deeper field uncertainty	Conservative currents; IRB/IEC risk management; enhanced monitoring	As for tES + seizure risk

Clinical populations also warrant special consideration. For example, sleep-disordered breathing (SDB) can introduce respiratory-related arousals that complicate closed-loop timing and safety, and insomnia may also increase the likelihood of awakenings with auditory stimulation. Screening for these conditions and diagnoses, conservative volume limits, and minimal-audibility titration are advisable.

#### 2.2.3 Methods to modulate rapid eye movement (REM) sleep

EEG theta waves (approximately 4-7 Hz) have remained a hallmark feature of REM sleep since the first sleep scoring manuals were released in the 1960's (Rechtschaffen A , eds). Recent work demonstrates that θ-frequency stimulation phase-locked within REM can increase theta power during REM without necessarily changing macro-architectural REM duration or probability (Harrington et al., 2021). Because arousal thresholds are lower in REM than N3, careful volume titration and conservative refractory periods are recommended to mitigate awakening risk (see Safety/Tolerability).

#### 2.2.4 Methods to hasten sleep onset and increase total sleep time

Methods focused on treating sleep onset insomnia attempt to hasten sleep onset with neurostimulation or biofeedback. Evidence of a consistent, reliable biofeedback technique is scarce, but biofeedback involving breath control has been suggested to hold promise (de Zambotti et al., 2019). In another study, neuro-feedback yielded either no effect or a negative effect as measured by self-report [Pittsburgh Sleep Quality Index (PSQI) scores] (Recio-Rodriguez et al., 2024). On the other hand, one recent study testing a novel handheld device to provide skin-temperature driven biofeedback of sleep onset improved Insomnia Severity Index scores in 40 individuals with insomnia (Ypsilanti et al., 2025).

Whereas biofeedback studies have yielded mixed results, recent neurostimulation work has begun to show consistent effects in accelerating sleep onset. Short duration, repetitive transcranial stimulation at 0.75 Hz decreased sleep onset latency more than control stimulation at higher frequencies (25 Hz) (Simons et al., 2024). Another promising study (*n* = 25 participants) reported that a tACS protocol reduced sleep onset latencies by 28% (6 min) (Ayanampudi et al., 2023). These approaches seek to entrain ongoing neural oscillations, biasing the brain's dominant activity toward lower-frequency bands—particularly δ and θ—that characterize the transition into and maintenance of sleep.

### 2.3 Safety, tolerability, and regulatory considerations

Most closed-loop sleep protocols aim to operate near sensory thresholds and below arousal levels; safety hinges on individual calibration, conservative refractory rules, home-use monitoring/logging, and exclusion of populations at elevated risk. Table 1 consolidates typical adverse events (AEs), practical contraindications, and monitoring guidance.

- Auditory (CLAS). Common AEs: brief micro-arousals if volume is too high; rare reports of next-day “ear fullness”; tinnitus risk considered low at near-threshold levels. *Guidance:* pre-sleep hearing thresholding; use of calibration nights in which stimuli are tuned to specific individuals' arousal thresholds (e.g., choosing a starting volume at perceptual threshold+10–20 dB and titrating during the overnight to determine maximum volumes that reliably do not induce arousals); hard caps on maximum levels; obeying strict refractory periods with duty cycling; and excluding individuals from the study who present with active otologic disease or unmanaged tinnitus.
- tES/tACS. Common AEs: tingling/itching/erythema at electrodes; rare headache; stimulation and switching artifacts that contaminate EEG for several seconds post-stimulation. *Guidance:* limit current density and session duration; exclude from study those with a history of seizure, implanted electronic devices, or skull defects; proper use of gel electrodes and skin checks.
- TMS. AEs: scalp discomfort, rare vasovagal responses; seizure risk is low with sleep-compatible parameters (but still non-zero). *Guidance:* lab-only use with medical oversight; pre-screening for seizure risk; avoiding at-home TMS.
- Thermal (cutaneous modulation). AEs: skin hot-spots or chills if control loops fail; theoretical risk for thermoregulatory illness if overdone. *Guidance:* small set-point deltas (< 1°C at skin), soft rate limits, redundant temperature sensing; exclude neuropathy with impaired thermal sensation, fever, or dysautonomia; caution in pregnancy and extremes of age.
- Focused ultrasound (fUS). AEs: transient paresthesia or warmth; safety depends on acoustic parameters (intensity, duty cycle) and skull heating. *Guidance:* remain well within diagnostic ultrasound intensity norms; use pre-validated transmit parameters; avoid at-home use.
- At-home ethics and privacy. Closed-loop devices should log stimuli/arousals, encrypt data, make algorithms and safety interlocks transparent, and allow user-controlled pause/stop functionality.

## 3 Outstanding questions

Despite significant progress in the development of neurostimulation techniques to enhance sleep (Table 2), several important questions remain. These range from straightforward questions of optimization (detector design, stimulation timing, equipment, etc.) to more fundamental unknowns about the limits of sleep modulation. Nevertheless in many cases the next steps are clear, with design implications for an optimal experimental system clearer still.

**Table 2:** Closed-loop sleep modulation paradigms: typical targets, timing, and outcomes.

**Paradigm**	**Primary target & timing rule**	**Typical sample**	**Key outcomes**	**Examples & Reviews**
CLAS (NREM)	SO up-state targeting; 50 ms pink-noise; fixed delay ~300-500 ms or phase-locked	Young adults; *n* per arm ~15-30; naps or overnight	SWA ↑; memory benefits meta-analytic *g*~0.3-1.1; mixed in older adults	Ngo et al., 2013; Ong et al., 2016; Wunderlin et al., 2021
tES/tACS (NREM)	SO-range tACS or slow oscillatory tDCS during N2/N3	Young adults; sample sizes similar to CLAS	SWA ↑; memory effects mixed; EEG artifact windows	Marshall et al., 2006; Dondé et al., 2021
Spindle-triggered	Stim during ongoing spindle or shortly pre-spindle	Young adults; *n*~20	Spindle density/coupling ↑; motor learning benefits in several (but not all) studies	Lustenberger et al., 2016; Choi et al., 2019; Choi and Jun, 2022; Baxter et al., 2023
REM theta paradigms	Theta-frequency pulse trains during REM epochs	Young adults; overnight	Theta power within REM ↑; macro-REM time not necessarily changed; arousal risk managed by titration	Harrington et al., 2021
Thermal (cutaneous)	Cyclic skin warming/cooling (Δ~0.4°C); rate-limited	Small adult samples; crossover designs	SWS ↑ overall; relative second-half SWS ↑↑; subjective sleep quality ↑	Raymann et al., 2008; Kräuchi et al., 2018

### 3.1 On augmenting slow waves, spindles and ripples with closed-loop neurostimulation

Although each of these oscillations can be stimulated directly, the literature to date has focused on slow wave stimulation given its widespread use, amenability to noninvasive techniques, and likely effectiveness at enhancing downstream physiology (spindles, ripples) as well.

- Detector design. Researchers have used different methods to detect endogenous oscillations to trigger stimulation (e.g., slow wave down state onset), from simple amplitude thresholding to more sophisticated signal processing and statistical approaches. What is the most sensitive detector design for slow waves, and how is it best fortified against false positives and signal artifact? The possible distinction between multiple types of slow waves naturally has important implications for detectors as well (see next bullet) (Siclari et al., 2014; Bernardi et al., 2018; Kim et al., 2019).
- Is the Slow Oscillation distinct from the δ-frequency Slow Wave? There is evidence that the spindle- and ripple-organizing slow oscillation (~0.5–1.25 Hz) is, in several respects, functionally and physiologically distinct from **δ** waves (1–3 Hz) that dominate N3. For example, one study found diverging effects on memory, with slow oscillations enhancing and δ slow waves suppressing memory (Kim et al., 2019). Another line of work describes two types of slow wave distinguished by their degree of widespread cortical synchronization and morphological characteristics (Bernardi et al., 2018), however results concerning differences in their central frequencies are mixed (Siclari et al., 2014; Bernardi et al., 2018). SOs and δ waves can co-occur during SWS (Achermann and Borbély, 1997). While practical dissociation between the two from EEG alone may be challenging, the conceptual distinction may matter significantly for protocol design (e.g., aiming to augment SOs for memory vs. boosting δ for increasing time in N3).
- Stimulation timing. While there is broad agreement that stimuli delivered during the slow wave's up state are optimal to increase slow wave activity (or its down state to suppress SWA), it is not yet known what precise timing is best, relative to a detected down state, and even whether this timing—within the confines of an up state—matters at all. The up state lasts several hundred milliseconds, and cortex is presumably excitable for this duration. Hence it could be that stimulation delivery anywhere within this time window is equally effective. It is also plausible that precise timing (phase-locking) on the order of < 10 ms *is* important. Techniques have ranged from fixed delays of approximately 400 ms post-detection, to subject-specific and even individual slow wave-specific timings derived from the estimated intrinsic period of the oscillation [e.g., by estimating the average delay between down and up states (Ngo et al., 2013), or performing online analytic signal-derived estimations of phase (Ong et al., 2016)]. What is the best signal processing and technical procedure to optimally time stimuli for maximal effect?
- Overnights or naps. Overnight sleep studies are costly and time-intensive. Fortunately, closed-loop slow wave augmentation and memory enhancement has been demonstrated successfully in short naps (Choi et al., 2019; Ong et al., 2016). Given this, and that relevant sleep physiology has been shown to be characteristically and functionally indistinguishable in naps compared to overnights (Mylonas et al., 2020), naps are increasingly becoming a preferred behavioral protocol for studies of sleep and sleep-enhancement. Yet nap studies are often done on well-rested sleepers, raising the issue that the full effects of neurostimulation may be masked by possible ceiling effects in these studies.
- Stimulation modality: auditory, electrical, magnetic. Early studies on neurostimulation-based slow wave augmentation used transcranial electrical stimulation (tES) for stimulus delivery (Marshall et al., 2006). In the mid-2010's, it became apparent that auditory stimulation could be as effective at boosting slow waves and enhancing memory (Ngo et al., 2013, 2015). Auditory stimulation's primary advantage may be that it does not block measurement of EEG signal during the stimulation period (and up to several seconds afterwards, depending on system parameters), as tES does. Transcranial magnetic stimulation (TMS) has also been used to effectively drive slow waves during NREM sleep (Massimini et al., 2007). TMS's primary advantage is spatial specificity, being able to target local areas of cortex instead of the global stimulation provided by auditory or electrical stimulation, yet current TMS systems are generally too bulky and expensive to leave the laboratory setting. Among the three techniques, there is no clear winner in terms of being significantly more effective at slow wave augmentation for memory enhancement, but practical considerations generally favor auditory stimulation given its safety, low cost, and portability. One key remaining question is whether any of the three technologies are stronger drivers of slow wave activity irrespective to any effect on memory.
  Temporal Interference (TI) techniques, in which two (or more) high-frequency (≳2 kHz) currents generate a low-frequency interference envelope at depth, in principle may enable non-invasive targeting of subcortical structures with minimal cutaneous sensation. There is early empirical evidence of TI-induced effects in humans (Missey et al., 2023; Wang et al., 2025; Acerbo et al., 2024). At the same time, a growing body of work has shown that as little as < 10% of current reaches the brain from tES, and that the magnitude of the electric fields is too small to directly elicit neuronal firing (Vöröslakos et al., 2018; Opitz et al., 2016). While subthreshold or indirect effects cannot be ruled out, it is still unclear whether this presents a viable approach to sleep neurostimulation.
- Upper bounds. Slow wave augmentation has been shown to be a self-limiting process with an inherent upper bound (Ngo et al., 2015). How much can this system be augmented before reaching its homeostatic limit?
- “No free lunch.” It is important to note that although tools that deepen N3 or boost SOs may yet improve “restorative efficiency,” they could also alter sleep architecture (e.g., change REM fraction/latency) in ways that are undesirable for some outcomes or populations. Future trials should track whether benefits on primary targets trade off against other stages or daytime functions. Given sleep's diverse roles in health, its restorative effects are likely multivariate, implying that personalized interventions will need to be tuned to optimize specific restorative systems according to individual characteristics and desired outcomes.

### 3.2 On modulating whole sleep states (NREM/REM on-demand)

Even more fundamental questions remain regarding this ambitious goal, but empirical evidence (reviewed above) suggests this is possible. Closed-loop modulation of peripheral and core body temperature (via e.g., cutaneous temperature modulation) in a sleep stage-dependent manner holds promise for enhancing NREM sleep (SWS). The same research suggests modulation by less than half a degree Celsius (°C) may be sufficient. How should this be timed relative to the sleeper's sleep state dynamics? What is the best effector to use? Can the system be miniaturized or made portable for field use by e.g., incorporating novel thermoelectric materials (Ballard et al., 2025; Osborn et al., 2024)?

These kinds of questions have historically been answered piecemeal by adapting existing technology to suit the experimenter's needs. Progress could be accelerated by a standardized device ecosystem designed from the start to support real-time sleep monitoring and closed-loop neurostimulation. Open-source access could further catalyze advances by undergirding this technology with a de-centralized developer community capable of sustaining support more reliably than individual companies or start-ups.

## 4 Design implications for sleep neurostimulation systems

Given the vast number of sleep neurostimulation protocols that have proven effective, engineering one bespoke solution is likely to be costly and inefficient. It is not yet clear which stimulation protocols will be most effective or desirable, and ultimately the choice may rest with which specific outcome is desired. However, in light of this uncertainty and the current state of the field, several conclusions can be drawn about what an optimal system design for applied research in the present should look like.

### 4.1 Modularity

Modularity should be the central tenet. The pub-sub design philosophy (applicable to both hardware and software design thinking) revolves around “reactive programming” using publisher-subscriber (pub-sub) paradigms; data sources (sensors), signal processing & compute modules, and data sinks (effectors) all communicate with each other using standard message formats to form a complex sum-of-parts system that is easy to modify and maintain. The field of robotics commonly relies on this type of sophisticated real-time architecture. Examples include “Robot Operating System” (ROS) (Quigley et al., 2009), “Lab Streaming Layer” (LSL) (Kothe et al., 2024), and ezmsg.^4^ Pub-sub structure allows distributed software to be fully encapsulated on distributed hardware—ideal for modularity. ROS, LSL, and ezmsg are all supported by an active community of users, developers, and researchers contributing to their open-source ecosystems, and already enjoy significant degrees of interoperability.

### 4.2 Sensors

A variety of sensor types and signal sources should be supported. Scalp EEG, actigraphy, and other physiological sensors [e.g., pulse plethysmography (PPG) to capture heart-rate variability] are among the most useful for monitoring sleep, and may become increasingly important for identifying artifact in forehead EEG signals as these wearable EEG devices become more common in the research and home-user community. Environmental sensors (e.g., ambient sound, light, etc.) and sensor feedback modules from effectors (e.g. a thermocouple to monitor a thermomodulating effector) may be needed to close the real-time loop and monitor system state. For all of this, a quality hardware abstraction layer—allowing the operating software to interface with sensor modules in a substrate independent manner—is critical. This allows consumption of data in a standardized format for e.g., time series data, in turn supporting a “plug-and-play” approach to system construction that is ideal for modular pub-sub design. Ideal sources include:

◇ Smart watches. Most have PPG sensors for heart rate (HR) and HRV, accelerometry for actigraphy, a microphone for sound/snore sensing, and position sensing.
◇ Smart rings. An Ōura smart ring uses PPG for HR/HRV and accelerometers for motion actigraphy. Open-source alternatives are also becoming available (Zhou et al., 2023).
◇ Smart pills. Ingestible “smart pills” are an emerging commercially available technology capable of monitoring core body temperature and providing real-time wireless telemetry (Mombers et al., 2016), a valuable metric for SWS stimulation in particular.
◇ EEG. The gold standard signal for sleep monitoring, and a necessity for fast-response, closed-loop stimulation protocols like slow wave stimulation via auditory stimuli (turnaround in less than a fifth of a second).

### 4.3 Reusable firmware/software

Programming languages like Rust are particularly well suited for implementing hardware abstraction layers (HAL) within a distributed architecture (Klabnik and Nichols, 2019). Its modular trait-based abstractions align naturally with the pub-sub design philosophy recommended here: each sensor or interface module can be expressed as an independent publisher or subscriber exposing a well-defined capability, while the compiler enforces type and thread safety across communicating modules for reliable operation. Rust was also explicitly designed to avoid race conditions in a distributed system, enabling precise and robust handling of timing and state across components. These HALs can easily be reused across platforms, accelerating design and prototyping as new technologies or sensor/effector units emerge. This portability will be increasingly important as the field continues to move toward scalable, multi-node closed-loop systems.

### 4.4 Effectors

Multiple effectors (for parallel intervention protocols) and effector types [for different delivery routes to the central nervous system (CNS); e.g., electrical, auditory, thermal] should be supported. Modular design will allow novel effector types to be added easily in the future, while also supporting multiple configurations in the present. Output effectors suggested by current understanding of the field include:

◇ Auditory. In-ear headphones, ambient speakers, bone conduction audio, etc.
◇ Electrical. e.g., transcranial direct- and alternating-current stimulation (tDCs/tACs), with or without Temporal Interfence (TI) techniques.
◇ Magnetic. Although transcranial magnetic stimulation (TMS) technology is presently too bulky for readily portable or wearable form factors, the ability to interface with such systems in a research laboratory setting would help pave the way for incorporation of miniaturized TMS in the future.
◇ Thermal. Heating/cooling mattresses or mattress pads are on-market examples; “smart clothes” with dynamically set, thermal-modulating fabrics represent the next generation of thermal modulators.
◇ IoT devices. Ambient light and temperature are essential environmental variables to capture in some sleep stimulation protocols, which can tie in to effectors as well (e.g., smart thermostats).
◇ Focused ultrasound (fUS). One emerging area of research has leveraged ultrasound emitters to target deep brain structures such as the thalamus (Caulfield et al., 2025; Murphy et al., 2022). fUS can also benefit from TI-like techniques, with intersecting beams of emitted energy producing the desired low-frequency modulation effect only at their point of intersection in 3-dimensional space; this allows for noninvasive deep targeting.

### 4.5 Compute

A modular design to compute power is also warranted. A system should be able to take advantage of distributed computing for scalable computation that spans cloud (remote servers), local (nearby computers), and wearable (embedded systems) compute platforms. Deploying the same pub-sub messaging service on each allows the system to be easily scaled down for lightweight, minimalist implementations (e.g., only a smart ring/watch, sans EEG, with a thermal smart-shirt effector) that are more cost effective, easier to deploy in the home or in operational settings, and well-suited for longitudinal studies/interventions. Conversely the system can be scaled up to include powerful A.I. and neural network decoders to translate EEG signals into real-time sleep staging and slow wave activity monitoring.

### 4.6 Next-generation power sources

**Figure 4:**
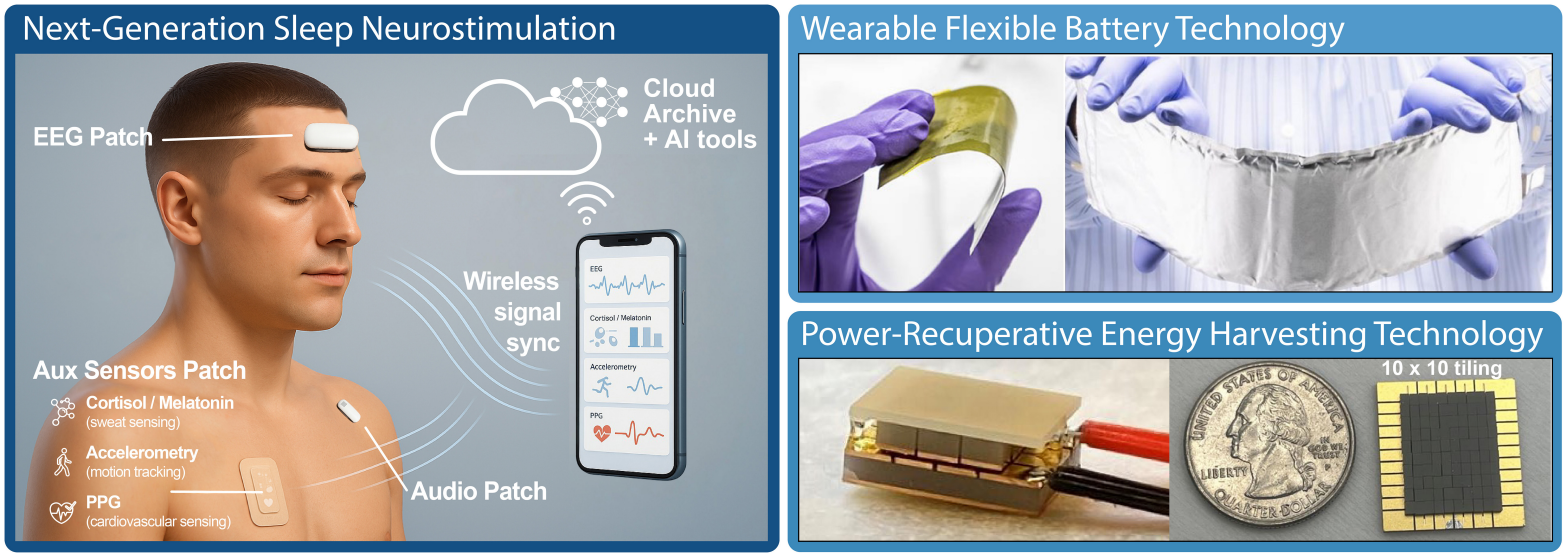
Emerging technologies for next-generation sleep neurostimulation. Modular wearable and attachable devices enable versatile applications in both research and healthcare settings. Advances in miniaturization, materials science, and manufacturing processes allow these systems to function continuously for extended periods, ranging from days to weeks. Such capabilities are essential for naturalistic longitudinal studies, enabling increased sample sizes and thereby facilitating more reliable, stable, and robust assessments of neurostimulation effects during sleep. **(Left)** Notional modular system for sleep assessment and stimulation control. **(Top Right)** Novel conformable battery technology ideal for sleep wearables (Logan et al., 2020). **(Bottom right)** Newly developed thin-film thermoelectric materials (Ballard et al., 2025; Osborn et al., 2024) enable power generation from heat gradients, making power recuperation through body heat possible in future wearable systems (Venkatasubramanian et al., 2007; Shen et al., 2025).

One way to effectively scale sample sizes to cohorts large enough for stable statistical evaluation of neurostimulation effects is to place renewed emphasis on at-home and longitudinal studies. For this, systems must be unobstrusive, lightweight, and able to operate for days to weeks at a time with minimal charging requirements. Several emerging technologies are available to support this (Figure 4), including flexible batteries with custom electrolyte chemistries and cell architectures specifically tailored to overcome electrode stability under mechanical stress, and long cycle life under repeated bending, torsion, and other deformations required for wearable energy storage (Logan et al., 2020). Energy harvesting techniques such as radiant body heat capture using novel thin-film thermoelectric materials can generate substantial power supplies without requiring wall-charging (Ballard et al., 2025; Osborn et al., 2024).

### 4.7 Rapid prototyping/iteration

Complete control over hardware design, data visibility and software access is so advantageous as to be almost a necessity: historically, the landscape of sleep devices has been fragmented and unreliable, with many consumer-oriented devices rotating on and off the market within a few years or less. Few offer complete “under-the-hood” access to firmware, software or data streams (especially in real time). Even many research-grade devices are generally proprietary black-box systems that provide limited or no access to data streams for system integration. Decoupling from this market allows for complete control over system design and therefore also the ability to rapidly iterate functional prototypes that are responsive to new research developments and emerging technologies. This may be the biggest remaining gap to close to advance sleep neurostimulation and enable the sleep device ecosystems envisioned here.

### 4.8 Extensible software

A well-documented and expertly supported scientific computing environment is important to support multiple teams and end users, and a thriving community of users/developers adds value to the ecosystem and ensures continuity of support. In general, the neural decoding and A.I. community has rallied around dynamic/interpreted languages like Python for these purposes. Python allows rapid implementation with fast turnaround and low overhead, avoiding reproduction of effort and reducing costs and design time. Extensive documentation and support lowers the barrier of entry for novice experimenters. ezmsg, ROS, and Lab Streaming Layer, for example, are extensively documented, designed explicitly for pub-sub modularity, and implemented in Python—well-aligned for use in the easy-to-use, highly configurable and extensible system envisioned.

### 4.9 Open hardware

**Figure 5:**
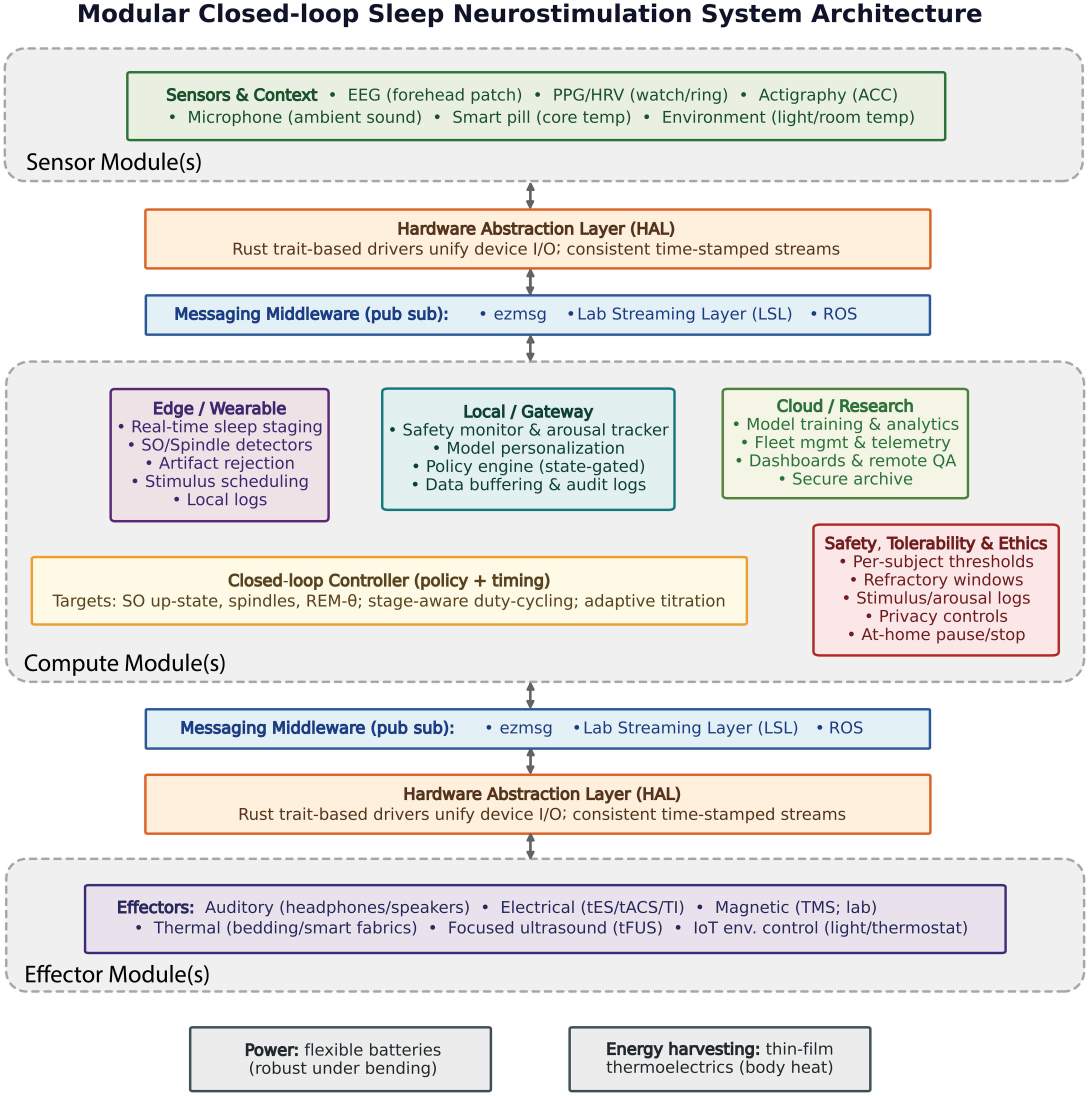
Proposed system architecture for next-generation sleep neurostimulation. The layout illustrates general design guidelines emphasizing modularity and interoperability rather than prescribing a fixed blueprint. System elements are organized into sensing, computing, and effector modules, linked through standardized pub-sub communication interfaces. Compute functions may operate on-device, on a mobile gateway, or via cloud resources, while sensor and effector modules can be flexibly attached or replaced through reusable software components.

A modular hardware and software ecosystem with the characteristics outlined above (overview in Figure 5) would allow diverse research groups to reduce development costs while benefiting from a highly customizable and user-friendly platform. Such an ecosystem would also promote open science, accelerating innovation and dissemination in the field of sleep neurostimulation. Encouragingly, several open-source hardware and software initiatives are beginning to emerge that align with these design goals (see Esfahani et al., 2024 for a recent overview). However, the vast majority of these efforts are software-focused, with relatively few providing openly available hardware schematics. Notable exceptions include platforms such as OpenBCI (OpenBCI, 2025; Fréy, 2016) and DCMini (Coon et al., 2025a,b), which offer open hardware architectures supported by community-contributed, open-source software. Open hardware will be crucial for enabling flexibility and modularity in design. Encouragingly for the sleep neurostimulation field, current trends suggest a growing momentum toward increasingly open and interoperable technology platforms.

## 5 Conclusions on advancing the field

The success that neurostimulation has enjoyed in enhancing sleep indicates there is good reason for optimism that sleep states and state transitions could be manipulated on-demand in the near future. Yet given the breadth of protocols established and remaining uncertainty regarding which protocol(s) will be able to support this vision, it is imperative to maintain flexible design in experimental systems. These systems should be modular, highly configurable, and designed from the start to be extensible. For maximum impact, they should rely as much as possible on freely available, actively developed and well-supported open scientific computing environments.

## Footnotes

1 Here, “closed loop” refers to stimulation that is mediated by brain activity processed in real time, thus closing the loop from brain to device and back to brain again.

2 Note that down states are used for detection instead of up states because their timing is much more clearly defined, and their amplitudes tend to be larger than up state amplitudes. Their timing is precise because the time spent in an up/down state is asymmetrically distributed: down states are brief (approximately 50–100 ms) and up states are prolonged (usually greater than approximately 400 ms).

3 as long as it is time-synchronized to endogenous slow waves.

4
https://ezmsg.readthedocs.io/en/latest/
